# Differential Impact of Olive Leaf Extract and Its Secoiridoid Components, Oleuropein Aglycone and Oleacin, on Adipogenic Differentiation and Proliferation of Bone Marrow Mesenchymal Stem Cells

**DOI:** 10.3390/ph19030353

**Published:** 2026-02-25

**Authors:** Chiara Giordani, Angelica Giuliani, Silvia Di Valerio, Tatiana Spadoni, Laura Graciotti, Sonia Bonacci, Antonio Domenico Procopio, Antonio Procopio, Maria Rita Rippo

**Affiliations:** 1 Clinic of Laboratory and Precision Medicine, IRCCS INRCA, 60129 Ancona, Italy; c.giordani@inrca.it (C.G.);; 2 Department of Clinical and Molecular Sciences, DISCLIMO, Università Politecnica delle Marche, 60126 Ancona, Italy; 3 Department of Biomedical Sciences and Public Health, Università Politecnica delle Marche, 60126 Ancona, Italy; 4 Department of Health Sciences, University “Magna Græcia” of Catanzaro, Campus Universitario “S. Venuta”, 88100 Catanzaro, Italy; 5 AGreen Food Research Center, University “Magna Græcia” of Catanzaro, 88100 Catanzaro, Italy

**Keywords:** mesenchymal stromal cells, adipogenesis, proliferation, olive leaf extract, oleacin, oleuropein aglycone

## Abstract

**Background/Objectives**: Bone marrow adipose tissue (BMAT) serves multiple physiological roles but accumulates with age, compromising skeletal health. This expansion is largely driven by an adipogenic drift of bone marrow mesenchymal stromal cells (BMSCs), shifting attention toward stromal cell fate regulation as a target to preserve bone marrow homeostasis. Preventing adipogenic commitment may be as relevant as directly inducing osteogenesis for maintaining a bone-permissive marrow microenvironment. Here, we investigated whether olive leaf extract (OLE) and its purified secoiridoid components, oleacin (OC) and oleuropein aglycone (OA), modulate the adipogenic differentiation and proliferative capacity of human BMSCs. **Methods**: Human BMSCs were induced to undergo adipogenic differentiation and treated with OLE, OC, or OA. Intracellular lipid accumulation and the expression of key adipogenic regulators were assessed. Proliferative capacity was evaluated under both maintenance and adipogenic conditions. **Results**: Under adipogenic conditions, OLE markedly reduced intracellular lipid accumulation and induced a coordinated downregulation of PPARγ, PLIN1, FABP4, ADIPOQ, LEP and the adipogenesis-associated miR-422a. In contrast, OC and OA exerted more selective and specific effects on biomarkers, indicating the partial and complementary modulation of adipogenic programs. Notably, OLE also increased BMSC proliferation under both maintenance and adipogenic conditions, suggesting the preservation of a less committed stromal cell pool. Although the relative contribution of enhanced proliferation versus the direct inhibition of adipogenic pathways cannot be fully disentangled, the combined molecular and functional data support a dual action of OLE on stromal cell fate. **Conclusions**: OLE limits adipogenic commitment while maintaining stromal cell proliferative competence, processes that are critically involved in BMAT expansion and bone marrow dysfunction. OC and OA contribute to OLE bioactivity deserving further investigation, particularly in combination, as potential modulators of BMAT expansion.

## 1. Introduction

Bone marrow mesenchymal stromal cells (BMSCs) are multipotent progenitors capable of differentiating into various mesodermal lineages, including adipocytes and osteoblasts. The equilibrium between these two pathways is critical for maintaining skeletal homeostasis and metabolic health. An imbalance in this differentiation potential contributes to the development of various chronic conditions. For example, during aging, bone marrow fat progressively accumulates, a phenomenon increasingly recognized as a contributor to age-related skeletal disorders, including osteoporosis [1,2]. While this adipogenic shift is often associated with negative outcomes for bone health, it is important to recognize that bone marrow adipocytes are not merely passive bystanders. Instead, they are metabolically active cells involved in key physiological functions, contributing to bone remodeling and hematopoiesis through the release of fatty acids, which serve as a vital energy source in the hypoxic ecosystem of the endosteal niche [3,4]. This dual role underscores the importance of the finely tuned regulation of adipogenic differentiation within the bone marrow microenvironment. Adipogenic differentiation is primarily governed by the transcription factor Peroxisome Proliferator-Activated Receptor Gamma (PPARγ), a master regulator that activates downstream targets such as fatty acid-binding protein 4 (FABP4), adiponectin (ADIPOQ), leptin (LEP), and perilipin 1 (PLIN1), all of which contribute to lipid accumulation and mature adipocyte function [5]. Peroxisome proliferator-activated receptor gamma coactivator-1α (PGC-1α) further modulates mitochondrial biogenesis and energy metabolism during adipogenesis [6].

While several strategies aimed at counteracting bone loss have focused on promoting osteoblast differentiation, increasing evidence indicates that preserving the functional competence and proliferative capacity of bone marrow stromal cells, while limiting their adipogenic drift, may represent an equally relevant—and perhaps more upstream—mechanism in the context of age-related alterations of the bone marrow microenvironment [7,8]. In osteoporotic and aged bone marrow, stromal cell dysfunction, exhaustion and reduced self-renewal capacity precede obvious defects in osteogenesis [9], suggesting that interventions acting on stromal fate decisions and progenitor maintenance could help to preserve a bone-permissive microenvironment. This conceptual shift—from direct lineage promotion to progenitor preservation—opens new avenues for therapeutic intervention targeting the bone marrow stromal compartment [10].

Within this framework, increasing attention has been directed toward biologically active compounds capable of modulating stromal cell behavior and lineage commitment. Olive leaf extract (OLE) is a polyphenol-rich preparation derived from *Olea europaea* that has demonstrated pleiotropic effects on inflammatory, metabolic, and oxidative stress pathways—all of which intersect with BMSC function and are dysregulated during aging. Although OLE has been extensively studied in non-skeletal contexts, its impact on bone marrow stromal cell fate regulation and adipogenic commitment remains insufficiently characterized. A diet enriched with olive oil or natural phytochemicals can modulate mesenchymal stem cell biology [11,12]. Among the emerging modulators of MSC differentiation, phytochemicals are being increasingly explored for their ability to influence lineage commitment by targeting key transcriptional and metabolic pathways [13]. OLE is a preparation rich in several phenolic compounds, primarily oleuropein, obtained from the *Olea europaea* leaves, and it has been shown to exert various bioactivities, including anti-inflammatory, antioxidant, and metabolic effects [14,15,16]. Although oleuropein and hydroxytyrosol are quantitatively the most abundant phenolic compounds in olive leaf extracts and have been widely studied for their anti-adipogenic and antioxidant properties [17,18,19], we decided to focus on the biologically intriguing secoiridoid derivatives oleacin (OC) and oleuropein aglycone (OA). These compounds are relevant bioactive constituents of extra virgin olive oil and are included among the compounds associated with the EFSA health claim on olive oil polyphenols. Despite their recognized relevance, they remain largely undercharacterized in BMSC biology. Moreover, their distinct structural and phenolic profiles suggest potentially differential biological activities, warranting direct comparative investigation.

Beyond their antioxidant properties, olive-derived secoiridoids have been reported to modulate inflammatory signaling, cellular stress responses across different experimental models [14] and lipid metabolism [20]. These processes are tightly interconnected and play a central role in regulating cell fate decisions, particularly under conditions of chronic inflammation and aging [14]. Given the tight interconnection between inflammatory signaling, lipid metabolism and the transcriptional regulation of adipogenesis, these observations provide a biological rationale to explore the impact of olive-derived phenolics on stromal cell fate decisions. However, despite these observations, no studies to date have comparatively assessed the effects of OLE, OC, and OA on the adipogenic differentiation of bone marrow-derived MSCs, nor their relative contribution within the complex phytochemical matrix. Importantly, while adipogenic commitment is commonly evaluated through well-established transcriptional and functional markers, emerging evidence indicates that this process is also fine-tuned by non-canonical molecular regulators. Among these, miR-422a has been proposed as one of the modulators of adipogenic transcriptional programs through the regulation of PPARγ-associated gene networks [21], but its role in bone marrow-derived MSCs adipogenesis remains largely unexplored. In this study, we investigate whether olive leaf extract and its bioactive phenolic constituents modulate bone marrow stromal cell fate by limiting adipogenic commitment and preserving proliferative capacity, integrating molecular and functional readouts relevant to bone marrow adiposity and dual.

## 2. Results

### 2.1. OLE, OC and OA Effect on BMSC Cell Viability

**Figure 1:**
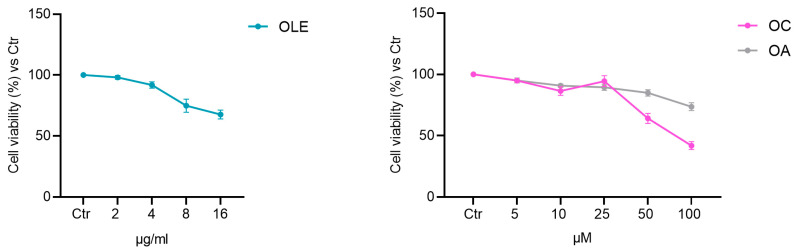
Effect of OLE, OC and OA on BMSC viability. Cells were treated with OLE (TPC from 0 to 16 μg/mL), OC and OA (from 0 to 100 μM) for 24 h. The results are presented as a percentage of cell viability, normalized to the viability of DMSO-treated cells (CTR), and shown as the mean value ± SD from three independent experiments.

OLE was prepared directly from freshly harvested olive leaves maceration in PBS and characterized in terms of total phenolic content (TPC from five preparations: 569.5 ± 22.142 µg/mL), as previously described [14]. Oleacin (OC) and oleuropein algycone (OA) were purified and chemically characterized as previously described [22,23]. The cytotoxicity of OLE, OC and OA was assessed in BMSC after 24 h treatment with the MTT assay (Figure 1). Further experiments were performed considering the concentration of each compound that would ensure at least 85% cell viability after treatment (5 μM OC and OA, 4 μg/mL OLE).

### 2.2. OLE, OC and OA Limit Adipogenic Commitment in BMSC

**Figure 2:**
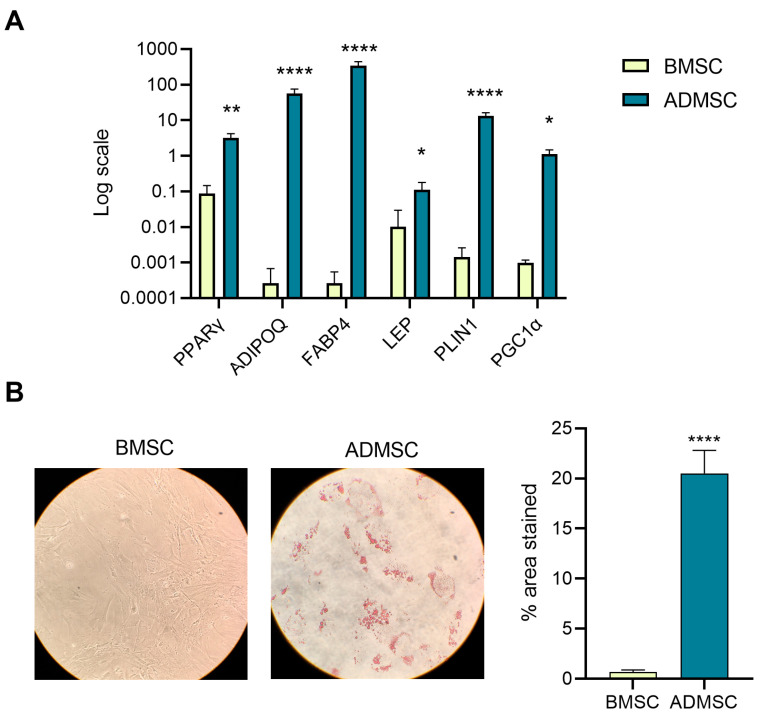
Adipogenic differentiation of BMSCs. (**A**) PPARγ, ADIPOQ, FABP4, LEP, PLIN1, PGC-1α mRNA expression of BMSCs cultured in adipogenic (ADMSC) or maintenance media (BMSC) for 14 days. Data are reported using a log scale according to the 2^−ΔΔCt^ method, using IPO8 as housekeeping. (**B**) Representative images of Oil Red O-stained ADMSCs and BMSCs. The histogram shows the mean of the percentage of area stained in three independent experiments analyzed with ImageJ (version 1.52a, Stuttgart, Germany). Paired *t* test, * *p* < 0.05, ** *p* < 0.01, **** *p* < 0.0001 vs. untreated BMSCs.

To evaluate the effect of OLE, OC and OA on lineage commitment, BMSCs were cultured under adipogenic conditions in the presence or absence of the compounds. Adipogenic induction resulted in marked lipid accumulation and an increased expression of adipocyte-associated markers compared to undifferentiated BMSCs (Figure 2).

**Figure 3:**
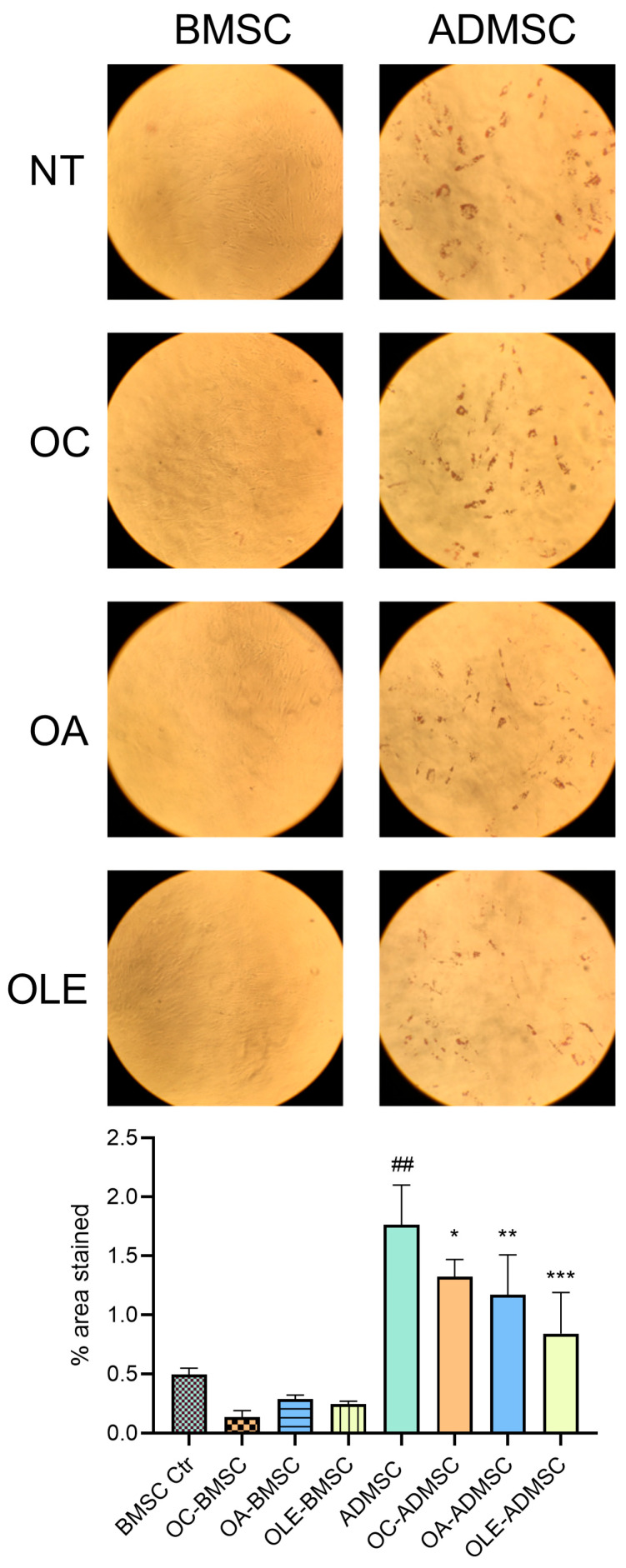
Effect of OC, OA and OLE on BMSC differentiation. Lipid droplet accumulation was quantified using Oil Red O staining in ADMSCs differentiated in the presence or absence of OC, OA and OLE and BMSCs. Representative images of Oil Red O-stained cells and histogram showing the mean percentage of area stained in three different photographs from three independent experiments, analyzed with ImageJ (version 1.52a, Stuttgart, Germany). Paired *t* test, ## *p* < 0.01 vs. BMSC Ctr; * *p* < 0.05, ** *p* < 0.01, *** *p* < 0.001 vs. ADMSC.

**Figure 4:**
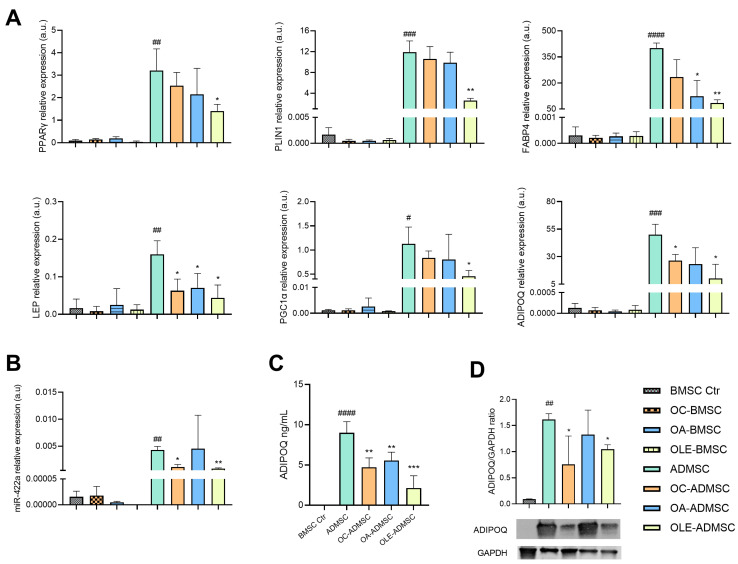
Effect of OLE, OC and OA on adipogenic marker expression in ADMSCs and BMSCs. BMSCs were differentiated into ADMSCs for 14 days in the presence or absence of OLE, OC and OA. (**A**) PPARγ, PLIN1, FABP4, LEP, PGC-1α, ADIPOQ mRNA expression was analyzed in ADMSC and compared with undifferentiated BMSCs under the same treatment. Data are shown as relative expression vs. not treated BMSCs (BMSC Ctr), according to the 2^−ΔΔCt^ method, using IPO8 as housekeeping. (**B**) miR-422a expression. Data are shown as relative expression vs. not treated BMSC Ctr, according to the 2^−ΔΔCt^ method, using RNU44 as housekeeping. (**C**) Concentration (ng/mL) of ADIPOQ in the culture medium of BMSCs and ADMSCs in the presence or absence of OLE, OC and OA. (**D**) Representative western blot analysis showing adiponectin protein expression, with GAPDH as loading control. The bands were quantified using ImageJ. All data are reported as fold change vs. BMSC Ctr. The results are expressed as mean ±SD from three independent biological replicates. Paired *t* test, # *p* < 0.05, ## *p* < 0.01, ### *p* < 0.001, #### *p* < 0.0001 vs. BMSC Ctr; * *p* < 0.05, ** *p* < 0.01, *** *p* < 0.001 vs. ADMSC.

Treatment with OLE significantly reduced intracellular lipid droplet accumulation, whereas OC and OA induced a more modest reduction (Figure 3). Consistently, OLE treatment led to a significant downregulation of adipogenic markers, including PPARγ, PLIN1, FABP4, ADIPOQ, LEP and PGC-1α, as well as miR-422a (Figure 4). In contrast, OC and OA selectively affected individual markers, with OC significantly reducing ADIPOQ, LEP and miR-422a expression, and OA reducing FABP4 and LEP expression. At the protein level, intracellular adiponectin expression was reduced by OLE and OC, while adiponectin release into the culture medium was decreased by all treatments, with the strongest effect observed in OLE-treated cells (Figure 4C,D, Table 1).

### 2.3. Proliferative Effect of OLE in BMSCs and Adipogenic-Induced BMSCs

**Figure 5:**
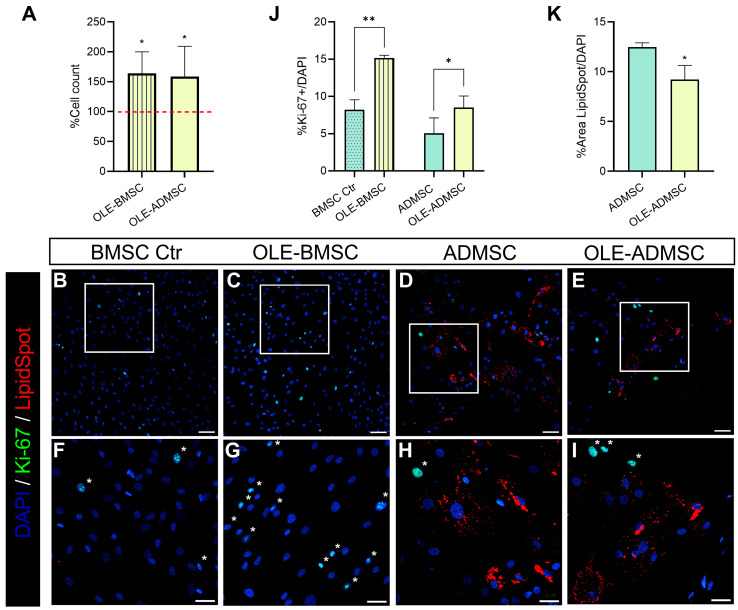
Immunofluorescence analysis of BMSCs and ADMSCs, treated or not with OLE for 14 days, to assess proliferative activity using Ki-67 staining. Image quantification was carried out using automated analysis across multiple microscopic fields. Representative images illustrate the typical staining pattern observed. Quantitative results are based on analysis of multiple microscopic fields per condition across independent experiments. (**A**) Cell counts of viable cells after treatment with OLE in BMSCs and ADMSCs. The bar graph shows the percentages relative to their respective untreated controls, indicated by the horizontal line set at 100%. (**B**–**E**) Cells were stained with an anti-Ki-67 antibody (green) to visualize proliferating nuclei, Hoechst 33342 (blue) (Molecular Probes, Eugene, OR, USA) to label all nuclei, and LipidSpot (red) to highlight lipid accumulation. Representative merged images are shown for each condition. (**F**–**I**) Higher magnifications of the boxed areas in panels (**B**–**E**), respectively, to emphasize cellular details. Asterisks indicate Ki-67-positive nuclei. Scale bars: (**B**–**E**) 100 µm; (**F**–**I**) 50 µm. (**J**) Quantification of proliferative activity. The histogram shows the percentage of Ki-67-positive nuclei relative to total nuclei (%Ki-67+/DAPI) across different conditions. (**K**) Quantification of lipid accumulation. The histogram shows the percentage of lipid-stained area relative to the total nuclear area. Data are presented as mean ± SD from three independent experiments. Paired *t* test, * *p* < 0.05, ** *p* < 0.01.

We observed an increased cell number in both BMSCs and ADMSCs treated with OLE compared to their respective untreated controls (Figure 5A), which led us to hypothesize a potential proliferative effect of the extract. To investigate this, we performed immunofluorescence analysis for Ki-67, a marker of cell proliferation, and LipidSpot (Biotium, Fremont, CA, USA), used to detect lipid accumulation at the end of the 14 days needed for differentiation (Figure 5B–I and Figure S1). The quantification of LipidSpot staining revealed no detectable signal in BMSCs and OLE-BMSCs, while it was clearly present in ADMSCs and significantly reduced in OLE-ADMSCs (Figure 5K). Ki-67 analysis confirmed a significant increase in proliferating nuclei in OLE-treated cells (Figure 5J). A visual inspection of the images indicated that Ki-67-positive nuclei appeared mainly in LipidSpot-negative populations (Figure 5D,E,H,I); however, this pattern was based on qualitative observation rather than quantitative analysis.

## 3. Discussion

In this study, we demonstrate that OLE and, to a lesser extent, its purified secoiridoid components OC and OA restrain adipogenic commitment in human BMSC while simultaneously—and unexpectedly—promoting their proliferative expansion. Notably, the olive leaf extract used in this study was obtained through an aqueous extraction process, avoiding organic solvents and allowing the recovery of a complex polyphenolic mixture from olive leaves [24,25], an agricultural byproduct, in line with the principles of circular economy. Compared with the isolation of individual compounds from extra virgin olive oil, this approach offers practical advantages in terms of extraction efficiency and cost, while preserving the combined bioactivity of the extract. All three treatments were well-tolerated by BMSCs at concentrations ensuring at least 85% cell viability, with the aqueous extraction method likely contributing to the low toxicity profile observed [26]. Under adipogenic conditions, OLE, OC, and OA significantly reduced intracellular lipid droplet accumulation, with OLE exerting the most pronounced effect. While the anti-adipogenic activity of olive-derived polyphenols has been previously reported [19], the present study extends these observations to BMSCs and provides a direct comparison between whole OLE and its individual secoiridoid components. Importantly, to our knowledge, this represents the first characterization of OC and OA in the context of human BMSC adipogenic differentiation. At the molecular level, OLE induced a coordinated downregulation of key adipogenic markers, including PPARγ, PGC1α, LEP, ADIPOQ, PLIN1 and FABP4, as well as the adipogenesis-associated microRNA miR-422a [21]. In this context, OC and OA have been shown to influence the expression of the key regulator of lipid homeostasis, including PPARγ, thereby attenuating adipogenic differentiation in various cellular systems [17,27,28]. Similarly, OLE has been associated with reduced lipid accumulation and the modulation of adipogenic markers in vivo [29,30]. In contrast, OC and OA exerted distinctive and partially overlapping effects: OC reduced ADIPOQ, LEP and miR-422a, while OA selectively inhibited FABP4 and LEP. This marker-specific interference provides mechanistic insight into their relative efficacies. Given the pro-lipogenic role of leptin [31] and the central function of FABP4 in intracellular fatty acids trafficking [32], OA’s dual targeting of these pathways could explain its moderately higher efficiency in limiting lipid droplets accumulation during BMSC differentiation compared to OC. Conversely, OC‘s suppression of ADIPOQ—a marker of terminal adipocyte differentiation [33]—suggests interference with late-stage adipogenic maturation. Notably, all three treatments reduced adiponectin secretion into culture medium, with OA potentially blocking release through post-translational mechanisms such as impaired folding or endoplasmic reticulum retention [34], an avenue requiring further investigation. Although the present study focused on representative adipogenic markers, the coordinated modulation of multiple components within the PPARγ regulatory network suggests pathway-level interference. Dedicated signaling pathway analyses will be necessary to identify upstream molecular targets involved in the OLE-mediated modulation of stromal cell fate. Overall, while previous studies have examined the bioactivity of individual olive-derived compounds, these findings underscore the superior efficacy of OLE compared to the individual compounds when used alone. This may be attributed to their lower phenolic content compared to OLE [14], indicating that a combination of various polyphenols may be necessary to exert synergistic effects, enhancing their regulatory action on stem cell adipogenic differentiation.

Beyond its effects on adipogenic differentiation, OLE exerted a previously unreported pro-proliferative effect on bone marrow stromal cells, as evidenced by increased Ki-67 positivity under both maintenance and adipogenic conditions, compared to their respective controls. In parallel, a significant reduction in lipid accumulation was observed in OLE-ADMSCs, suggesting that proliferation and lineage commitment are jointly modulated. These findings support the hypothesis that OLE favors the maintenance and expansion of an undifferentiated BMSC pool, potentially delaying or suppressing their transition into adipocytes. With the current experimental design, it is not possible to definitively distinguish whether the reduced adipogenic differentiation reflects a direct inhibition of adipogenic pathways or an indirect consequence of enhanced stromal cell proliferation, leading to a higher proportion of undifferentiated cells. However, the coordinated downregulation of multiple adipogenic regulators observed at the molecular level (Figure 4 and Figure 5) supports the notion that OLE acts on lineage-related mechanisms beyond a mere dilution effect.

Overall, these results highlight the potential of OLE as a multifunctional natural compound capable of supporting interventions targeting bone marrow, not only by reducing adiposity but also by promoting BMSC self-renewal and proliferative capacity. This dual action is particularly relevant in the context of aging and chronic inflammatory conditions, in which bone marrow stromal cells exhibit reduced proliferative capacity and functional exhaustion. Indeed, MSC isolated from older donors exhibit a reduced number of self-renewing cells, reflecting an age-related decline in their proliferative potential [35]. This decline impairs their ability to contribute to bone regeneration and to maintain the hematopoietic stem cell niche [36]. Therefore, the proliferative stimulus induced by OLE may help to preserve or restore BMSC functionality in these contexts, indirectly sustaining both skeletal repair mechanisms and hematopoiesis.

Future studies in aging and bone loss will be required to determine whether the preservation of stromal cell competence translates into maintained osteogenic potential and improved skeletal outcomes.

## 4. Materials and Methods

### 4.1. Cell Culture

Human mesenchymal stromal cells from the bone marrow (BMSC) of a 30-year-old female donor were purchased from PromoCell (Heidelberg, Germany) and cultured in αMEM, supplemented with 10% FBS and 1% penicillin/streptomycin (all from EuroClone, Milano, Italy). The cells were seeded at a density of 5 × 10^3^ cells/cm^2^ in T75 flasks (Corning Incorporated, Life Sciences, New York, NY, USA), maintained at 37 °C in a humidified atmosphere with 5% CO_2_ incubator (HERAcell 150i CO_2_ Incubator, Thermo Scientific, Waltham, MA, USA). The culture medium was changed with complete fresh medium every 48 h, and cells were trypsinized when they achieved approximately 80% confluence in culture. The cell line has been checked for Mycoplasma (Luna^®^ Universal qPCR Master Mix; BioLabs Inc., Cambridge, MA, USA).

### 4.2. MSC In Vitro Differentiation

The adipogenic differentiation of BMSCs (ADMSCs) was obtained using αMEM, supplemented with 10% FBS, 0.5 µM dexamethasone, 5 µg/mL human insulin, 0.45 mM 3-isobutyl-methylxanthine (IBMX), and 0.2 mM indomethacin (Sigma-Aldrich, Saint Louis, MO, USA). Medium was changed every three days. After 14 days, both ADMSCs and BMSCs were stained with Oil Red O to test adipogenic differentiation.

### 4.3. Oil Red O Staining

Oil Red O staining was used to evaluate lipid droplet accumulation after 14 days in BMSCs and ADMSCs. BMSCs and ADMSCs cells were washed with PBS (Dulbecco’s Phosphate Buffered Saline; EuroClone) and fixed with 4% paraformaldehyde (*w*/*v*) for 5 min at room temperature. A 0.5% (*w*/*v*), Oil Red O stock solution was prepared by dissolving Oil Red O in isopropanol and then filtered through a 0.22 µm membrane filter (EuroClone). A working solution was then obtained by diluting the stock solution to 0.3% (*v*/*v*) in distilled water before use. Fixed cells were covered with the staining solution for 20 min at room temperature in the dark. Later, cells were washed two times with PBS to remove the excess dye. The staining cells images were acquired under the light microscopy (Nikon Eclipse TS100, Minato, Japan). Three independent photographs from three independent experiments were selected and analyzed using the freely available imaging software ImageJ (version 1.52a, Stuttgart, Germany). The analysis was conducted by threshold-converting the 8-bit red–green–blue image into a binary picture, containing only pixels representative of lipid droplets. The resulting mean of the percentage of area stained was compared to control images (non-differentiated cells).

### 4.4. Cell Treatments

Cell treatments were performed as previously reported by our group [14]. Briefly, BMSCs and ADMSCs were exposed to an aqueous olive leaf extract (OLE)—extracted in PBS from the olive leaves of *Olea europea* L., cultivar Leccino in Marche region (Italy)—and two of its main bioactive compounds: 3,4-DHPEA-EDA (oleacin, OC) and 3,4-DHPEA-EA (oleuropein aglycone, OA), directly purified from OLE. All of them were obtained and chemically characterized prior to use [14,22,23].

### 4.5. Cell Viability Assay

Cell viability was assessed through the MTT (3-(4,5-dimethylthiazol-2-yl)-2,5-diphenyltetrazolium bromide), a colorimetric assay related to cell metabolic activity. Cells were seeded for 24 h in 96-well plates (Cell Culture Cluster, Tissue Culture Treated #3599; Corning Incorporated Corning, New York, NY, USA) at a density of 5 × 10^3^ cells/cm^2^ and then treated with different amounts of OLE (different doses in terms of TPC) or with different concentrations of oleacin (OC) and oleuropein aglycone (OA) for 24 h. In detail, the OC and OA concentration range tested was 0–100 µM, whereas OLE’s was from 0 to 16 µg/mL. DMSO-treated cells were used as the control group (CTR). MTT work solution (5 mg/mL) was added in each well (10 µL of MTT solution every 100 µL of medium) and incubated at 37 °C for 4 h (HERAcell 150i CO_2_ Incubator; Thermo Scientific). After the removal of culture medium with MTT solution from each well, the insoluble blue/purple formazan salts produced in living cells were solubilized by adding 100 µL DMSO (dimethyl sulfoxide, D5879; Sigma-aldrich, Saint Louis, MO, USA). The absorbance was measured at OD 540 nm using a microplate reader (NB-12-0035 Microplate Reader, NeoBiotech Co., Seoul, Republic of Korea). Data were expressed as a percentage of viability, according to the equation C:100% = T:X, where T and C represent the mean OD of treated cells and untreated cells (control group, CTR), respectively. In accordance with the previous equation, the CTR group was considered as 100% of viability. The following experiments were carried out using compound concentrations that maintained at least 85% cell viability after OLE, OC and OA treatments.

### 4.6. RNA Isolation and mRNA Expression by RT-qPCR

Total RNA was isolated using the Total RNA Purification kit (NORGEN Biotek corp., Wilmington, DE, USA) following the manufacturer’s guidelines. RNA was stored at −80 °C (CryoCube F570; Eppendorf, Hamburg, Germany) until use. The total amount of RNA was defined by spectrophotometric quantification with NanoDrop One (Thermo Fisher Scientific) and reverse transcribed using PrimeScript^TM^ RT reagent kit with gDNA Eraser (Takara Bio Inc., Tokyo, Japan), following manufacturer’s instructions. mRNA expression was evaluated by RT-qPCR using TB Green^®^ Premix Ex Taq^TM^ (Tli RNaseH Plus (Takara Bio Inc., Tokyo, Japan) using a Rotor-Gene Q (Qiagen, Hilden, Germany) apparatus. The following primers sequences (written 5′-3′) were all from Sigma-Aldrich: IPO8 (FW:CGTTCCTCCTGAGACTCTGC, RV:GAATGCCCACTGCATAGGTT); ADIPOQ (FW:CCTAAGGGAGACATCGGTGA, RV:GTAAAGCGAATGGGCATGTT); FABP4 (FW: TCACCTGGAAGACAGCTCCT, RV: AAGCCCACTCCCACTTCTTT); LEPTIN (FW:GGCTTTGGCCCTATCTTTTC; RV:CCAAACCGGTGACTTTCTGT); PGC-1α (FW: ACAACACTTACAAGCCAAACCA, RV: GCCTGCAGTTCCAGAGAGTT); PLIN1 (FW:GAAAAGATCCCCGCCCTCC, RV:CTGATGCTGTTTCTGGCACTG); PPARγ (FW: AGCCTCATGAAGAGCCTTCCA, RV: ACCCTTGCATCCTTCACAAGC). The mRNA expression levels of the gene of interest were calculated using a logarithmic scale according to the 2^−∆∆Ct^ method, with IPO8 used as endogenous housekeeping control.

### 4.7. miRNA Expression by RT-qPCR

MiRNA expression was measured by RT-qPCR using the TaqMan^TM^ MicroRNA Reverse Transcription kit and TaqMan^®^ MicroRNA Assays (all from Applied Biosystems by Thermo Fisher Scientific), following the manufacturer’s instructions. The following miRNAs sequences were all from Applied biosystems (Waltham, MA, USA): RNU44 (RT:001094/PN4440887 (20X), TM:001094/PN4440887 (20X); hsa-miR-422a (RT:002297/PN4427975 (5X), TM:002297/PN4427975 (20X)). The miRNA expression was estimated using relative expression according to the 2^−∆∆Ct^ method with RNU44 as housekeeping control.

### 4.8. Western Blotting

Cell pellets were lysed using RIPA lysis buffer (10 mM Tris, pH 7.2, 150 mM NaCl, 1.0% Triton X-100, 0.1% SDS, 5 mM EDTA, pH 8.0) adding protease and phosphatase inhibitor cocktail (Roche Applied Science, Indianapolis, IN, USA) at the time of lysis. Bradford (B6916 Sigma-aldrich) colorimetric protein assay and a microplate reader (NB-12-0035 NeoBiotech) were used to quantify the total protein concentration in each sample. After that, proteins (25 ng) were analyzed with SDS-PAGE using Precast 4–15% gels (Mini-PROTEAN^®^ TGX™ Precast Protein Gels, Bio-Rad, Laboratories, Inc., Hercules, CA, USA) and transferred to a nitrocellulose membrane (Bio-Rad, Laboratories, Inc., Hercules, CA, USA). Next, the EveryBlot Blocking Buffer (Cat. #12010020 Bio-Rad Laboratories, Inc., Hercules, CA, USA) was used to block the not specific sites of the membrane, which was then incubated overnight at 4 °C with primary antibodies. Mouse Anti-Adiponectin (#AMab22554, Clone:19F1; abcam, Cambridge, UK) and Rabbit GAPDH (#2118S, Clone:14C10; Cell Signaling, Danvers, MA, USA) were used as primary antibodies. Horseradish peroxidase (HR)-conjugated antibodies, anti-mouse or anti-rabbit (The Jackson Laboratory, Bar Harbor, ME, USA), were used as secondary antibodies. Protein bands were visualized by using the Clarity ECL chemiluminescence substrate (Bio-Rad Laboratories, Inc., Hercules, CA, USA) with Uvitec Imager (UVItec, Cambridge, UK) and then quantified using ImageJ software (version 1.52a).

### 4.9. ELISA

BMSCs and ADMSCs supernatants were collected after each treatment, centrifuged and stored at −80 °C until use. Human Adiponectin ELISA Kit (Cat. No. AG-45A-0001YEK-KI01, AdipoGen Life Sciences, San Diego, CA, USA) was used to measure the concentration of human ADIPOQ release in accordance with the manufacturer’s protocol. The assay range was 0.5–32 ng/mL and the sensitivity (limit of detection) was 100 pg/mL. The kit included a 96-well plate precoated with a monoclonal antibody specific for adiponectin, along with all the necessary solutions. Finally, the OD at 450 nm was measured within 30 min to complete the experiment.

### 4.10. Immunofluorescence

BMSCs and ADMSCs treated or not with OLE were seeded at a density of 5 × 10^3^/cm^2^ on polylysine-coated coverslips (Poly-L-lysine solution; Sigma-aldrich). Cells were washed twice with PBS and then fixed in paraformaldehyde 4% (PFA) in PBS for 20 min at room temperature. After three washes in PBS, cells were incubated in blocking buffer (5% BSA and 0.3% Triton X-100 in PBS), followed by incubation with anti-Ki-67 antibody (1:50, #MB67, sc-56319 Santa Cruz, Dallas, TX, USA) in 1% BSA and 0.3% Triton X-100 in PBS overnight at 4 °C in a humidified chamber. Secondary antibody incubation was performed at room temperature for 1 h (1:100; Alexa Fluor^®^ 488-conjugated AffiniPure Goat anti-rabbit IgG H + L, cat. no. 111-545-003, Jackson Immunoresearch Laboratories Inc., Ely, UK) in the same buffer as the primary antibody. After three washes in PBS, the slides were incubated with 1X LipidSpot 610 (Biotium, Fremont, CA, USA) for 10 min in the dark. After that, nuclei were stained with Hoechst 33342 (10 mg/mL solution in water; Molecular Probes, Eugene, OR, USA) for 5 min in the dark, and the cover slips were mounted with Vectashield mounting medium (H-1000, Vector Laboratories, Inc., Newark, NJ, USA). Images were acquired with an inverted microscope Eclipse Ti2-E supplied with an AX confocal system (Nikon, Minato, Japan) at ×20 magnification. 

For each condition, Ki-67 and LipidSpot quantification were performed on 10 microscopic fields per independent experiment, corresponding to an average of approximately 80 nuclei per field. The analysis was repeated across three independent experiments. All analyses were carried out using an automated FIJI macro to minimize operator bias, and thresholding and segmentation parameters were kept constant across all images. 

To ensure standardized and unbiased image analysis, a custom macro was developed in FIJI (ImageJ) for batch processing, enabling objective and reproducible quantification of Ki-67-positive nuclei and LipidSpot staining. Images were first converted to 8-bit grayscale, followed by automatic threshold adjustment using Otsu’s method [37]. For Ki-67 analysis, binary masks were generated to segment nuclei (DAPI-stained) and Ki-67-positive areas (green). Nuclei were classified as positive if they contained any detectable Ki-67 signal, regardless of the number or intensity of individual Ki-67 foci. This was achieved by overlaying the Ki-67 and DAPI masks, ensuring that each nucleus was counted as either positive or negative, rather than summing individual Ki-67 foci within a nucleus. The percentage of Ki-67-positive nuclei was then calculated relative to the total number of nuclei per field. For lipid accumulation analysis, the stained area was quantified rather than individual signal foci, given the diffuse and heterogeneous nature of the LipidSpot staining. The total lipid-stained area was measured using the same thresholding approach, and the percentage of stained area was calculated relative to the nuclear area.

### 4.11. Statistical Analysis

Data were analyzed and visualized using IBM SPSS Statistics for Windows, version 25.0 (IBM Corp., Armonk, NY, USA) and GraphPad Prism, version 7.0 (GraphPad Software, San Diego, CA, USA). Results are presented as mean ± standard deviation (SD) from at least three independent experiments. Student’s t test was applied to determine differences between samples. All tests were two-tailed, and *p*-values < 0.05 were considered statistically significant.

## 5. Limitations

This study has some limitations that should be considered. BMSCs were derived from a single donor, and therefore, donor-to-donor variability was not addressed. In addition, the experimental design was limited to an in vitro setting. Although our findings indicate that OLE limits adipogenic commitment and preserves stromal proliferative capacity, the current design does not allow definitive discrimination between the direct inhibition of differentiation and indirect effects secondary to enhanced proliferation. Additional single-cell analyses would help to clarify this aspect. Furthermore, in vivo studies in established models of aging-related bone loss will be necessary to determine whether these effects translate into reduced bone marrow adiposity and preserved skeletal function. Given the well-recognized contribution of adipogenic drift to bone deterioration during aging, such validation will be important to define the physiological relevance of these observations.

## 6. Conclusions

This study demonstrates that OLE and its bioactive secoiridoid components modulate adipogenic commitment and proliferative capacity in human BMSCs. While OC and OA exert selective and complementary effects on adipogenic regulators, the whole extract displays a broader activity, limiting adipogenic differentiation while preserving stromal cell proliferation. These findings support the concept that the integrated phytochemical matrix of OLE is required to effectively regulate stromal cell fate. Given that adipogenic drift and reduced stromal cell competence are hallmarks of aging and age-related bone marrow dysfunction, the present data provide a cellular framework linking olive-derived phytochemicals to mechanisms relevant to aging. Although further studies are needed to assess in vivo relevance and translational potential, this work highlights OLE as a promising phytochemical-based modulator of aging-associated alterations in stromal cell biology.

## Figures and Tables

**Table 1:** Effect of OLE, OC and OA in ADMSCs. “↓” indicates a statistically significant difference vs. ADMSC. “↓” *p* < 0.05, “↓↓” *p* < 0.01, “↓↓↓” *p* < 0.001, “-“ not significant.

	PPARγ	PLIN-1	FABP4	PGC-1α	LEP	ADIPOQ mRNA	ADIPOQ ELISA	ADIPOQ WB	miR-422a
OC	-	-	-	-	↓	↓	↓↓	↓	↓
OA	-	-	↓	-	↓	-	↓↓	-	-
OLE	↓	↓↓	↓↓	↓	↓	↓	↓↓↓	↓	↓↓

## Data Availability

Data are contained within the article.

## Supplementary Materials

The following supporting information can be downloaded at: https://www.mdpi.com/article/10.3390/ph19030353/s1, Figure S1: Representative fluorescence images showing nuclear staining with the proliferative marker Ki-67 conjugated with Alexa Fluor 488 (green), lipid droplets stained with LipidSpot Cy5 (magenta) and DAPI (blue). The scale bar is shown in the images and corresponds to 100 μm. Panels A, B, C, and D correspond to Figure 5B, Figure 5C, Figure 5D and Figure 5E, respectively. Merge images are presented in the main figure.

## Abbreviations

The following abbreviations are used in this manuscript:

ADIPOQ	adiponectin
ADMSC	adipogenic differentiation of mesenchymal stromal cells
αMEM	alpha minimum essential medium
BMSC	bone marrow mesenchymal stromal cell
BMAT	bone marrow adipose tissue
CTR	control
DMSO	dimethyl sulfoxide
ELISA	enzyme-linked immunosorbent assay
EVOO	extra virgin olive oil
FABP4	fatty acid-binding protein 4
FBS	fetal bovine serum
IBMX	3-isobutyl-1-methylxanthine
LEP	leptin
miR-422a	microRNA-422a
MTT	3-(4,5-dimethylthiazol-2-yl)-2,5-diphenyltetrazolium bromide
OA	oleuropein aglycone
OC	oleacin
OLE	olive leaf extract
PBS	phosphate-buffered saline
PFA	paraformaldehyde
PGC-1α	peroxisome proliferator-activated receptor gamma coactivator 1-alpha
PLIN1	perilipin 1
PPARγ	peroxisome proliferator-activated receptor gamma
qPCR	quantitative polymerase chain reaction
SD	standard deviation
WB	Western blotting
