# Supplementary figures and images for: Differential Impact of Olive Leaf Extract and Its Secoiridoid Components, Oleuropein Aglycone and Oleacin, on Adipogenic Differentiation and Proliferation of Bone Marrow Mesenchymal Stem Cells
Source: Pharmaceuticals (Basel). 2026 Feb 25;19(3):353. doi: 10.3390/ph19030353 (PMC13028832; doi:10.3390/ph19030353)

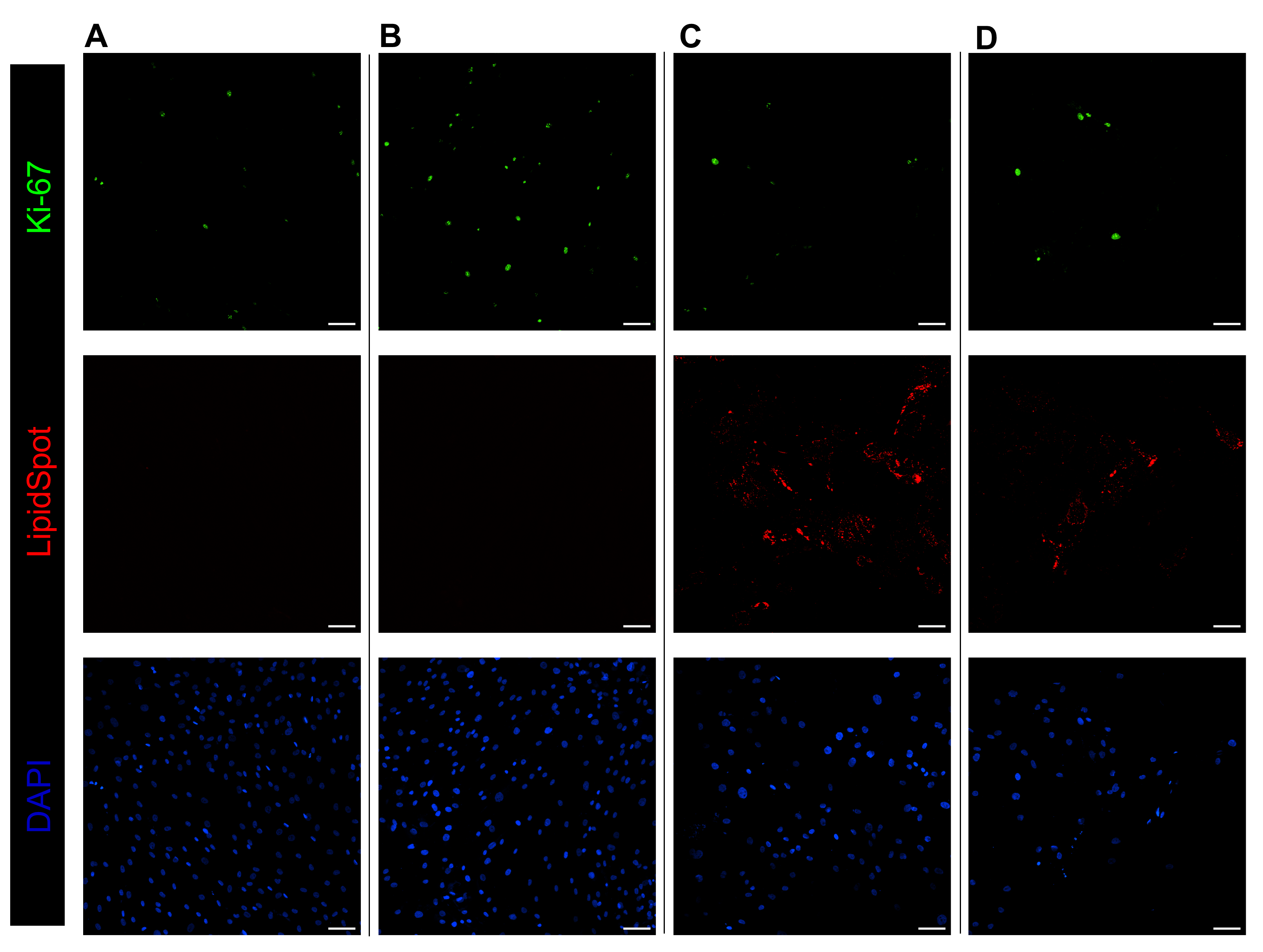

Supplement: Supplementary file 1 [file pharmaceuticals-19-00353-s001.zip › pharmaceuticals-4161070-supplementary.tif]
